# Identifying clusters of precipitation for the Brazilian Legal Amazon based on magnitude of trends and its correlation with sea surface temperature

**DOI:** 10.1038/s41598-024-63583-x

**Published:** 2024-06-18

**Authors:** Rodrigo Martins Moreira, Bruno César dos Santos, Trent Biggs, Fernando de Sales, Stefan Sieber

**Affiliations:** 1 Geomatics and Statistics Laboratory, Environmental Engineering Research Group, Department of Environmental Engineering, Graduate Program in Environmental Sciences, Federal University of Rondônia, Ji-Paraná, RO 76801-974 Brazil; 2 Department of Geography, San Diego State University, San Diego, CA 92182 USA; 3 Department of Environmental Sciences, Federal University of São Carlos, São Carlos, SP 13565-905 Brazil; 4 Leibniz-Zentrum für Agrarlandschaftsforschung (ZALF) e. V., 15374 Müncheberg, Germany; 5 Department of Agricultural Economics, Humboldt Universität zu Berlin, 10099 Berlin, Germany

**Keywords:** Climate sciences, Climate change, Hydrology

## Abstract

Prioritizing watershed management interventions relies on delineating homogeneous precipitation regions. In this study, we identify these regions in the Brazilian Legal Amazon based on the magnitude of Sen’s Slope trends using annual precipitation data from September to August, employing the Google Earth Engine platform. Utilizing the silhouette method, we determine four distinct clusters representing zones of homogeneous precipitation patterns. Cluster 0 exhibits a significant median increase in precipitation of 3.20 mm year^−1^ over the period from 1981 to 2020. Cluster 1 shows a notable increase of 8.13 mm year^−1^, while Clusters 2 and 3 demonstrate reductions in precipitation of − 1.61 mm year^−1^ and − 3.87 mm year^−1^, respectively, all statistically significant. Notably, the region known as the arc of deforestation falls within Cluster 2, indicating a concerning trend of reduced precipitation. Additionally, our analysis reveals significant correlations between Sea Surface Temperature (SST) in various oceanic regions and precipitation patterns over the Brazilian Legal Amazon. Particularly noteworthy is the strong positive correlation with SST in the South Atlantic, while negative correlations are observed with SST in the South Pacific and North Atlantic. These findings provide valuable insights for enhancing climate adaptation strategies in the Brazilian Legal Amazon region.

## Introduction

Precipitation is one of the major climatic variables of the hydrological cycle, being responsible for the regulation and equilibrium of tropical forests and presenting high spatial and temporal variability. The Brazilian Legal Amazon (BLA), the largest tropical forest in the world, is key to understanding regional and global hydrological cycles under climate change. Precipitation affects irrigation and water availability for urban areas and its surplus may cause floods and soil loss^1^. Urban and traditional communities of the BLA area are highly dependent on rainfall, causing them to be highly threatened by climate change^2^. Identifying trends in precipitation is important to design strategies to tackle these communities’ low climatic adaptability.

Various climate models provided by the Intergovernmental Panel on Climate Change (IPCC) are utilized to depict forthcoming trends globally, employing distinct climate change scenarios, causing adverse impacts with several losses and damages to nature and people, where vulnerable communities are the most affected^3^. A recent investigation examining climate change in South America with the aid of these scenarios foresees a decrease in precipitation by the year 2040, particularly in the northern and northeastern regions of South America, particularly during September, October, and November. This projected decrease in rainfall is anticipated to render the climate drier in these areas, rendering them more vulnerable to forest fires^4^.

Studies on climate change are typically conducted on a global or regional scale. In either case, they often concentrate on regions with a wide range of climatic conditions^5^. Sub-daily, daily, pentad, monthly seasonal and annual surface climate variables (such as minimum and maximum precipitation) have substantial regional gradients in terms of their temporal variability throughout the Amazon. Therefore, it is necessary to apply robust statistical techniques to estimate the magnitude of trends that consider spatial gradients^6,7^.

Several studies have explored regionalization and clustering methods to regionalize study areas into smaller and statistically similar zones for analysis. Identifying precipitation zones and regionalizing its patterns is key for decision making of strategic planning of areas such agriculture, urban infrastructure, water availability and climatic adaptability. The most common methods to aggregate climate variables include watershed boundaries, geopolitical boundaries, major atmospheric circulation mechanisms, elevation and simple rectangles over the study area^8^.

The BLA has been the subject of several studies regarding spatial and temporal trends of precipitation. Mu et al.^9^ analyzed a time series of precipitation for the southwestern region of the BLA at the monthly and seasonal time scales and compared their drought events and temporal trends. Haghtalab et al.^10^ analyzed a time series from 1982 to 2018 for the Amazon Basin and have deployed several precipitation indices and analyzed their trends using the Mann–Kendall test. Mu and Jones^11^ studied precipitation and deforestation age for the BLA and concluded that regions with old ages of deforestation show negative trends in dry season rainfall. Nevertheless, to the researchers’ knowledge, no study has used the Sen’s Slope magnitude of change to identify regions of change in precipitation in the BLA. We applied a pixel-by-pixel magnitude of change of trend analysis using Sen’s Slope and identified vulnerable zones for drought and flooding. This enhancement would add value to local analysis for an improved assessment of changes in patterns of precipitation, key for data-driven decision-making and to allocate strategic resources that contribute to traditional community’s climate change adaptability.

Regionalization of precipitation in the BLA was a challenge until recently due to lack of a reliable network of rain gauges, which are sparse, and the amount of missing data becomes also a constraint and its large spatial scale, which would require a great computer processing capacity. Datasets on rainfall derived from satellite imagery are now available in Google Earth Engine cloud computing platform. One of the many datasets available is the Climate Hazards group InfraRed Precipitation with Stations (CHIRPS), a state-of-the-art quasi-global rainfall estimation product available at daily to seasonal time scales, with a spatial resolution of 0.05°, and a 1981 to near real-time period of record^12^. When compared to other datasets, using GPCC as benchmark, such as ARC2, CFS-Reanalysis, CHIRP, CMORPH, CPC-Unified, ECMWF, PERSIANNE, RFE2, TAMSAT, TRMM-RT7, and TRMM-V7, CHIRPS has higher correlation and lower systematic errors (bias) and mean absolute errors than other precipitation datasets using GPCC as benchmark^13^.

Seasonality of the atmospheric systems that act on the Amazon region such as the Intertropical Convergence Zone (ITCZ), the South Atlantic Convergence Zone (SACZ), and the South American Low-Level Jet and Anticyclone System (SASA). The ITCZ is a region near the equator where the trade winds from the northern and southern hemispheres converge, causing uplift of warm and moist air, which are intricately linked to the dynamics of northeastern trade winds and their impact on both oceanic and continental masses. The migration of the ITCZ is driven by fluctuations in surface temperatures of the Atlantic Ocean, leading to temperature differentials with the South American landmass and shaping the characteristic seasonal rainfall cycle of the Amazon region. This results in heavy rainfall in the Amazon basin, particularly in the northern part of the region, during the wet season^14^. The SACZ is a region of convergence of the trade winds over the Atlantic Ocean, located south of the equator. It is responsible for a secondary rainy season in the southern Amazon region during the austral summer^14^. The SACZ is also associated with the formation of convective clouds and thunderstorms. The SASA is a circulation pattern that includes a low-level jet stream and a high-pressure anticyclone. The low-level jet stream transports moisture from the Amazon basin to the southern part of South America, while the high-pressure anticyclone inhibits convection and causes dry conditions over the central Amazon region during the austral winter^15^. Mu et al.^9^ concludes that during the drought years, moisture input from the west and south of the State of Rondônia (southwestern of the BLA) and the Atlantic Ocean drastically decreased compared to the long-term norm (1981–2018). The 2015-26 El-Niño drove to an unprecedent drought in the Amazon rainforest, with consequence of mostly extreme drought conditions concentrated into in the northeast region^16^. Lucas et al.^17^ studied precipitation indices in the Xingu watershed (east of the BLA) and its research shows a decrease trend in precipitation in the central area and an increase in the northern part of the basin; while the number of consecutive dry days presents a positive trend along most part of the basin, concentrated in its central part.

This study's objective is to regionalize the trends of increase and decrease in precipitation for the BLA. The trends will be identified using Sen’s Slope, which was calculated using the Google Earth Engine cloud computing platform. We then regionalize the precipitation based on the Sen’s Slope for annual and monthly time scales. We also test correlations between SST and rainfall in each region.

## Material and methods

### Study area

The BLA was institutionalized by Federal Law No. 1806, 1953 which encompassed the states of Acre, Amapá, Amazonas, Pará, Rondônia, Roraima, Tocantins, Mato Grosso, and a portion of Maranhão. The total area of the BLA is 5 million km^2^.

**Figure 1:**
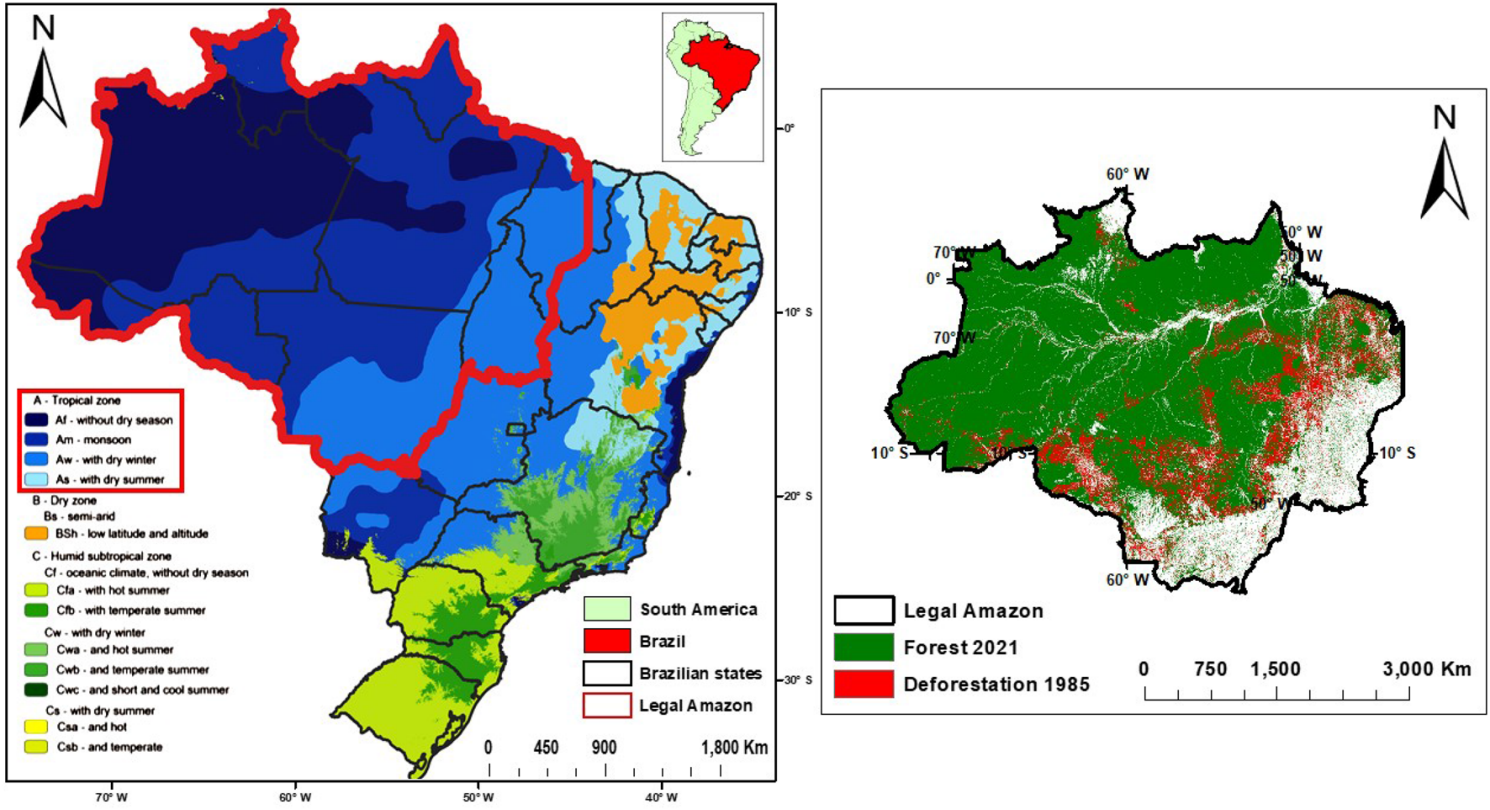
Area of study displaying the Köppen-Geiger classification for Brazil^23^, the Brazilian Legal Amazon in red (left) and deforestation since 1985 and remnant forest in 2021 (right).

On a local, regional, and global level, various physical and dynamical phenomena impact the BLA’s varying climate. This region exhibits an “A” type climate, typical of tropical woods, without a cold season, with average minimum temperatures over 18 °C, according to the Köppen classification. Three sub climatic regions, rainy equatorial (“Af”), tropical monsoon (“Am”), and tropical wet and dry (“Aw”), can be distinguished within the area, as seen in Fig. 1. Among the tropical classifications, the Af climate denotes regions characterized by consistently high temperatures and abundant rainfall throughout the year, without a distinct dry season; Am classification are areas experiencing a monsoonal climate, featuring high temperatures year-round with pronounced wet and dry seasons, typically associated with the seasonal shift in monsoon winds; Aw climate are regions with high temperatures throughout the year but distinct dry winters, whereas the As climate reflects similar temperature patterns but with dry summers. The development of the South American Monsoon System, which is affected by the sea surface temperature close to the equator, is primarily responsible for the seasonality of rainfall and the quick change between wet and dry seasons^18,19^. In general, intra- and interannual rainfall variability in the Amazon is controlled by sea surface temperatures in the Pacific and the Tropical Atlantic^20^. Figure 1 was generated using QGis version 3.34^21^. All other figures were generated using RStudio version 2023.12.1+402^22^ (RStudio Team, 2024).

### CHIRPS precipitation data

For the historical analysis of precipitation data from the Brazilian Legal Amazon, classification and rainfall trends tools were used. The methods were applied pixel-by-pixel in pentad and monthly accumulates of precipitation.

CHIRPS (Climate Hazards Group InfraRed Precipitation with Station data), deployed by Funk et al.^12^, combine satellite-based precipitation estimates with ground-based rain gauge observations. CHIRPS is designed to address the limitations of each data source when used alone, by leveraging the strengths of both. Specifically, satellite-based estimates are used to fill in the gaps where rain gauges are sparse or non-existent, while rain gauges provide ground truth data to improve the accuracy of the satellite-based estimates. The resulting product provides high-quality, high-resolution with 0.05° precipitation data that can be used for a variety of applications, from drought monitoring to flood forecasting. The methodology has been described in detail in Funk et al.^12^ and has been widely used and validated in various regions of the world.

CHIRPS data were obtained in matrix format with Tagged Image File (TIF) extension, including annual precipitation from 1981 to 2020, in 40 images. To post-process the spatial data to create manuscript quality figures, free and open-source software RStudio^22^ was used.

Supplementary material 2 presents boxplots of precipitation (mm pentad^−1^) for each cluster with significance tests (α = 0.01), and supplementary material 3 presents Time series of precipitation (mm pentad^−1^) for each cluster from 1981 to 2020.

### Sea surface temperature

The Advanced Very High-Resolution Radiometer (AVHRR) Pathfinder Sea Surface Temperature (SST), available in the Google Earth Engine dataset catalog, is a high resolution, long-term climate data record of global satellite SST, created and maintained by the NOAA National Centers for Environmental Information (NCEI)^24^. The AVHRR instruments mounted on NOAA polar-orbiting satellites dating back to 1981 are used to produce these SST readings at a resolution of about 4 km. The most recent version of the PF SST products is the AVHRR Pathfinder Version 5.3 (PFV53). This is a development toward the upcoming Version 6 (PFV6) data set. The SST was collected from 0 to 30 degrees of latitude.

### Sen’s slope pixel-by-pixel magnitude of trend analysis

Pixel-by-pixel Sen’s Slope magnitude of trends were calculated in Google Earth Engine using the reducer function ee.Reducer.sensSlope for ee.ImageCollection time series. In GEE, ee.Reducer.sensSlope() is a method used to compute the sensitivity (slope) of a linear model fit to a series of image bands over time. This reducer is particularly useful for analyzing time series data, such as satellite imagery, to detect and quantify trends or changes over time. The sensitivity slope represents the rate of change in the pixel values of the image bands over the specified time period. By applying the ee.Reducer.sensSlope() to an image collection, a linear model is fitted to the pixel values of each band over time using ordinary least squares regression. The resulting slope of this linear model represents the magnitude of change per unit time for each pixel and band. The significance was calculated in RStudio^22^ with the spatialEco (version 2.0-2)^24^ package using the stack of the yearly sum of precipitation for the water year. CHIRPS data were used as input to the Sen slope test in a yearly temporal scale. We aggregated according to the water year, from September to August^25,26^. In the various methods applied for this purpose, the normality of the dataset is a prerequisite, being highly sensitive to outliers. To overcome this limiting factor, it is necessary to apply a more robust test adapted to non-parametric data, such as Sen’s Slope (SS)^27^, aimed at identifying magnitudes in time series trends.

$$SS = Median\left\{ {\left[ {\left( {\frac{{x_{i} - x_{j} }}{i - j}} \right)_{j = 1}^{j = n - 1} } \right]_{i = j + 1}^{i = n} } \right\}$$

### K-means clustering

After identifying the magnitude of trends over the study area using the Sen’s Slope method. The clusters were identified. First, the Silhouette Analysis was deployed to identify the number of clusters. The clusters were calculated for different time periods, being (a) identification of clusters using the trends for the entire time series; (b) identification of clusters for each month of the time series, from January to December. For each time period, the Sen’s Slope was calculated to identify the magnitude of trend and the Silhouette analysis was deployed to identify the number of clusters.

### K-means clustering

To identify the optimum number of clusters, the Silhouette score was used. The silhouette score measures how effectively samples are clustered with other samples that are like them to assess the quality of clusters produced by clustering algorithms like K-Means^28^. Each sample of various clusters receives a Silhouette score. The following distances must be determined for each observation/data point belonging to each cluster to calculate the Silhouette score for that point.

The clustering process was deployed in Google Earth Engine using the ee.Clusterer.wekaKMeans(). The basic principle of k-means clustering is to identify clusters with the goal of minimizing total intra-cluster variation, also referred to as total within-cluster variation. In this clustering method, *n* objects are divided into *k* clusters, and each object is assigned to the cluster that has the closest mean. The maximum number of distinct clusters produced by this method is *k*. It is necessary to compute the best number of clusters *k* that will result in the greatest separation (distance) because it is not known a priori. Various k-means algorithms are available. The common approach is the Hartigan-Wong algorithm^28^, which sums the squared distances between items and the associated centroid in Euclidean space to determine the total within-cluster variation:

$$W\left( {C_{k} } \right) = \mathop \sum \limits_{i = 2}^{n} \left( {x_{i} - \mu_{k} } \right)^{2}$$

where, $x_{i}$ is a value point assigned to the cluster $C_{k}$; $\mu_{k}$ is the mean value of the group belonging to the cluster $C_{k}$. Each observation $x_{i}$ is given a cluster assignment that minimizes the sum of squares (SS) distance between the observation and the cluster center $\mu_{k}$. The following equation is applied to define the overall within-cluster variation.

$$tot.withiness = \mathop \sum \limits_{k = 1}^{k} W\left( {C_{k} } \right) = \mathop \sum \limits_{k = 1}^{k} \mathop \sum \limits_{{x_{i} \in C_{k} }}^{{}} \left( {x_{i} - \mu_{k} } \right)^{2}$$

The total within-cluster sum of squares measures the goodness of fit of the clustering and the optimum value is the smallest.

Supplementary material 1 presents the clusters for each month and the number of clusters identified for each month.

### Pettitt’s test for change-point detection

Pettitt’s test^29^ tests the null hypothesis (H0) that a variable follows one or more distributions that have the same location parameter (no change), against the alternative hypothesis (H1) that a change point exists. The non-parametric statistic of this test is presented as:

$$K_{T} = \left| {U_{t,T} } \right|$$,where:

$$U_{t,T} = \mathop \sum \limits_{i = 1}^{t} \mathop \sum \limits_{j = t + 1}^{T} signal\left( {x_{j} - x_{i} } \right)$$

The change-point of the time series is found at $K_{T}$, provided that the statistic is significant. The significance probability of $K_{T}$ is approximated for p ≤ 0.05 with:

$$p \cong \left( {\frac{{ - 6K_{T}^{2} }}{{T^{3} + T^{2} }}} \right)$$

Thus, given a significance level *α*, if *p* < *α*, the null hypothesis, H_0_ that the two distributions are equal is rejected.

## Results

### Description of time series of precipitation for each cluster and significance between each time series

**Figure 2:**
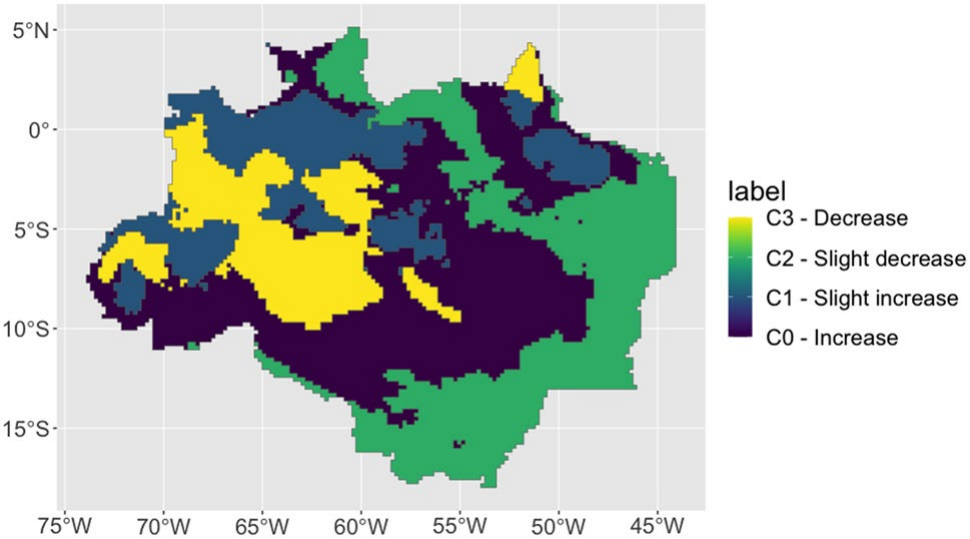
Sen’s slope magnitude of trend for precipitation (mm year^−1^) overlapped with the identified clusters on the top panel. The lower panel displays the identified clusters using the K-means method.

K-means clustering, and silhouette analysis resulted in 4 mapped clusters. Cluster 2 has the lowest and cluster 3 the highest median pentad precipitation (Table 1). Cluster 2 has the lowest and cluster 3 presents the highest. Cluster 0 presents the lowest and cluster 2 presents the highest standard deviation in pentad precipitation (Fig. 2). Medians were statistically significantly different using the Kruskall-Wallis's test.

**Table 1:** Descriptive statistics for each cluster calculated from the time series of precipitation (mm pentad^−1^) from 1981 to 2020.

Variable	Mean	Std. Dev.	Min	Pctl. 25	Pctl. 75	Max
ts_cluster_0	29	18	3.2	13	42	95
ts_cluster_1	36	14	7.1	25	46	103
ts_cluster_2	24	16	2	9.8	35	89
ts_cluster_3	36	16	5.1	22	49	101

Figure 2 suggests that there are four distinct patterns in the trends of the CHIRPS pixel-by-pixel time series data for the BLA. In this sense, four different clusters are identified in the BLA, where cluster 0 presents an increase in precipitation, cluster 1 presents a slight increase in precipitation, cluster 2 presents a slight decrease in precipiation and cluster 3 presents a decrease in precipitation.

In this analysis, a “slight increase” or “slight reduction” in precipitation refers to changes in precipitation levels that exhibit a relatively small magnitude of trend over the specified time period. This determination is based on the magnitude of the observed trend rather than solely on statistical significance. A “slight increase” denotes a positive trend indicating a gradual rise in precipitation levels, while a “slight reduction” indicates a negative trend signifying a modest decrease in precipitation levels.

### Silhouette analysis to identify the number of clusters

**Figure 3:**
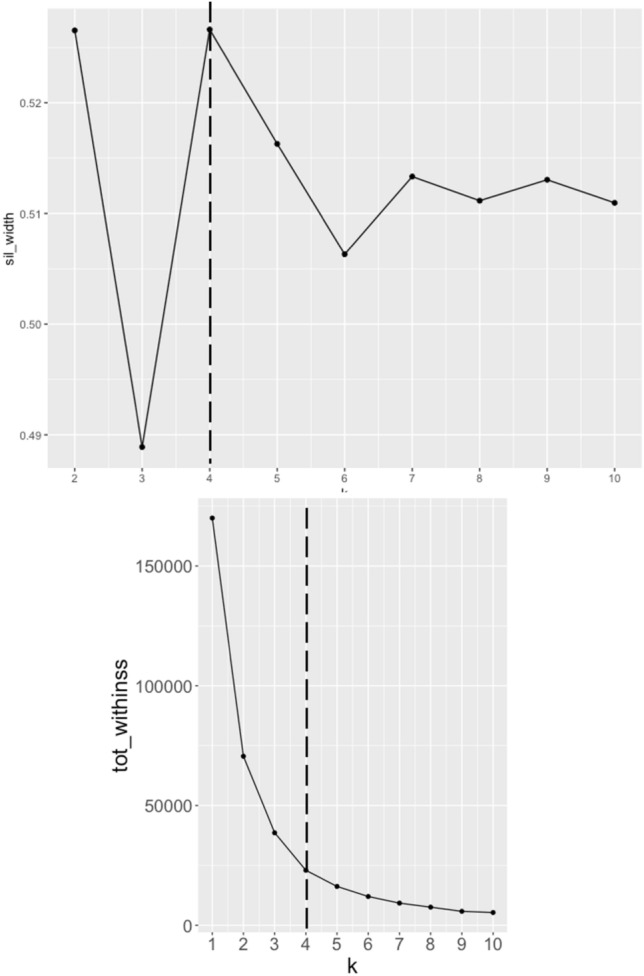
Silhouette and Elbow analysis to identify the number of clusters.

In the analyzed dataset, the optimum number of clusters is determined to be 4, the silhouette analysis results will show that the samples within each cluster have a high silhouette coefficient, indicating that they are similar to one another and well-matched to their own cluster. The silhouette coefficients between the clusters will be low, indicating that the samples in different clusters are dissimilar, as shown in Fig. 3.

### Clusters identified using the magnitude of the trend based on Sen’s Slope

In the analysis of clusters, we first present the calculation of the Sen’s Slope applied over the mean time series for all the pixels of each cluster from 1981 to 2020.

Our results in Table 2 shows Cluster 0 indicating a mean increase of 3.10 mm year^−1^ (p < 0.01, α = 0.01) in precipitation from 1981 to 2020. Cluster 1 indicates a mean increase of 8.5 mm year^−1^ (p < 0.01, α = 0.01). Cluster 2 suggests a mean reduction of 2.2 mm year^−1^ (p < 0.01, α = 0.01). And finally, Cluster 3 indicates a mean reduction in precipitation of − 4.4 mm year^−1^ (p < 0.01, α = 0.01).

**Table 2:** Descriptive statistics for the boundaries of each cluster.

Variable	# of pixels	Mean	Std. Dev.	Min	Pctl. 25	Pctl. 75	Max
SS_cluster_0_water_year	60,255	3.1	3.9	− 16	0.72	5.7	23
SS_cluster_1_water_year	28,082	8.5	3.8	− 3.3	5.8	11	28
SS_cluster_2_water_year	47,985	2.2	5	− 17	− 1.2	5.1	24
SS_cluster_3_water_year	27,339	− 4.4	5.9	− 26	− 8.8	0.32	10

These values were calculated using the output raster of the calculation of the Sen’s Slope, using as input the CHIRPS pentad dataset from 1981 to 2020.

**Figure 4:**
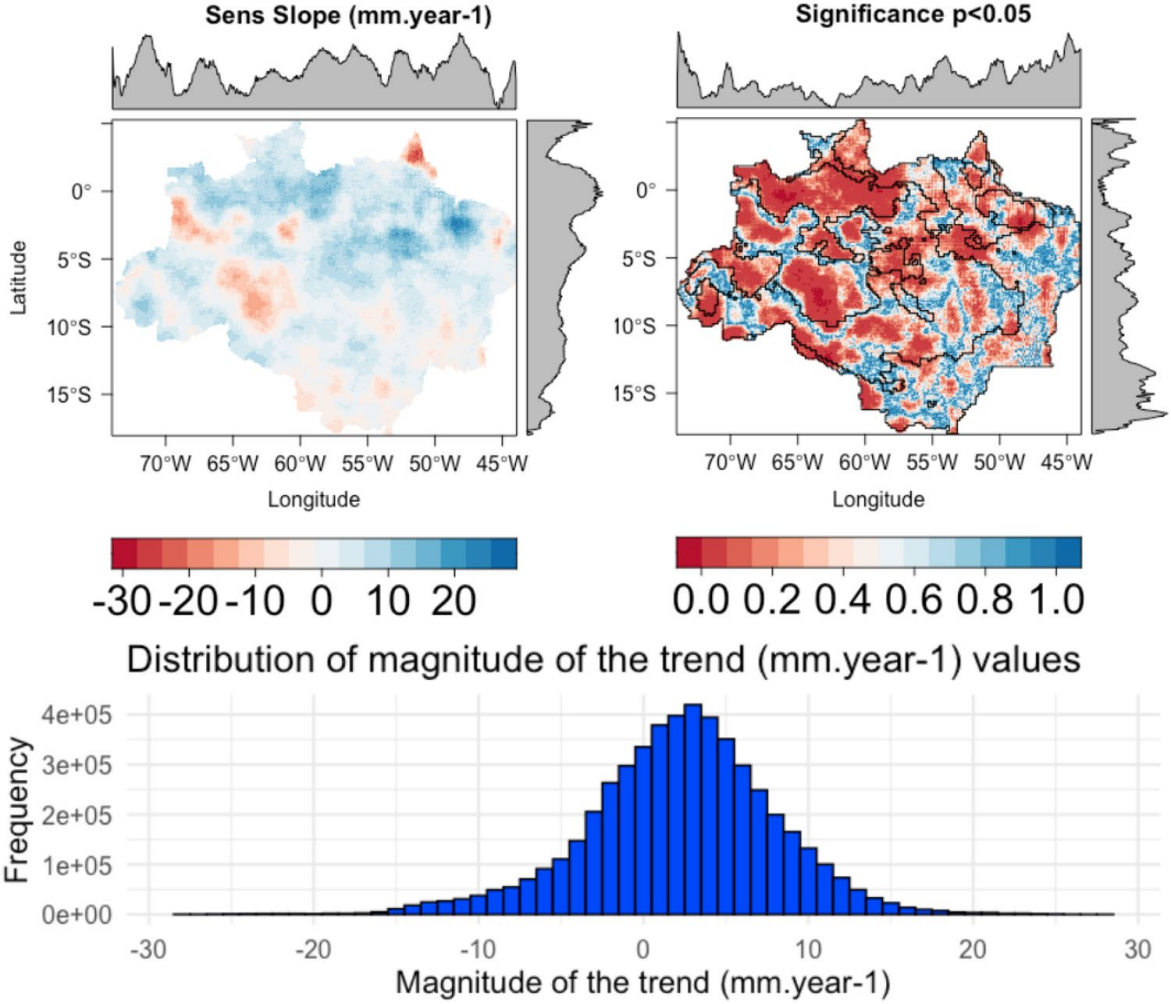
Pixel-by-pixel spatial distribution of Sen’s Slope (precipitation in mm year^−1^) for the Brazilian Legal Amazon, calculated using CHIRPS pentad time series aggregated for the water year of September through August from 1981 to 2020. Significance of the Sen’s Slope with p-value colors ranging from red to blue, low to high values, respectively (α = 0.05). Black lines display clusters zones. Histogram with the Sen’s Slope distribution.

Regionalization of precipitation in the Amazon rainforest can provide insight into the spatial and temporal variability and distribution of rainfall in similar regimen regions. K-means clustering has been used to study regionalization of precipitation in the Amazon, as seen in Fig. 4. These results corroborate with our findings, since applying K-means clustering over the pixel-by-pixel calculation of Sen’s Slope for the Brazilian legal Amazon also found four main areas of precipitation variability. All these studies demonstrate the potential of k-means clustering as a useful tool to identify patterns of precipitation in the Amazon. However, it is important to note that the results of these studies may vary depending on the dataset used, the period of analysis and the methods applied.

Pixel-by-pixel magnitude of the rainfall trend (Fig. 4) shows the longitudinal and latitudinal distribution of rainfall trends for the Amazon region. Clusters with significant positive and negative rainfall trends were found in the Amazonian regions. Significant negative trends occurred in the north and central-west clusters. Significant positive trends were observed mainly in the central and part of the north region of the BLA. Silva Junior^30^ found similar results for Sen’s Slope over the BLA, also finding negative values up to − 20 mm. Figure 1 of the supplementary material presents the clusters for each month.

**Figure 5:**
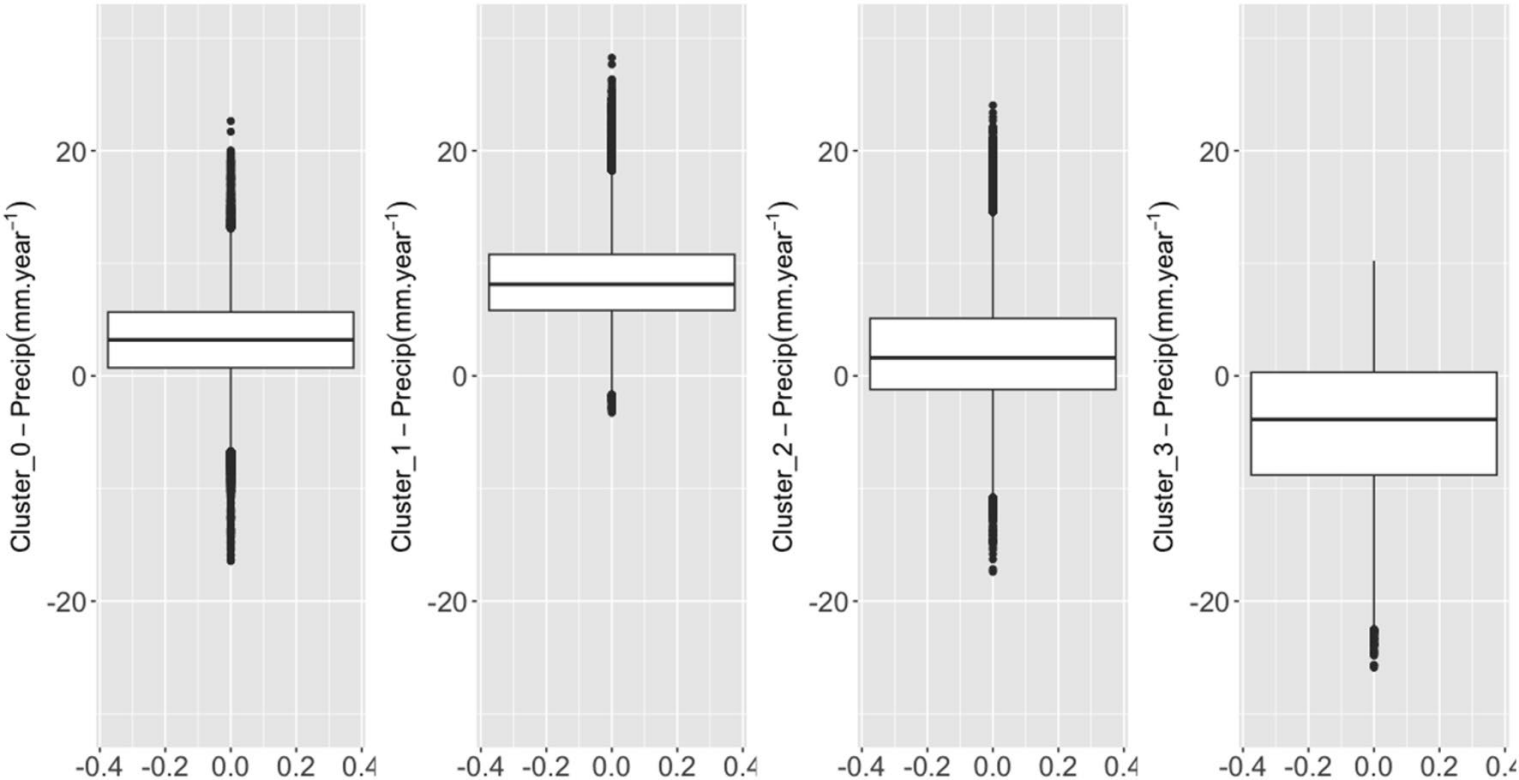
Boxplots for the Sen’s Slope precipitation (mm year^−1^) values presenting the magnitude of change in trends calculated using CHIRPS pentad time series aggregated for the water year of September through August from 1981 to 2020.

Figure 5 shows the distribution of the pixel values per cluster, by applying the Wilcoxon test, the null hypothesis is rejected in the pairwise comparison between clusters.

Much of the Amazon region presents a regionalization of rainfall within climate patterns, followed by areas above the standard and finally, small areas below the climate pattern. Clusters 0 and 3 presented the highest and lowest values of the Sen Slope (Table 3).

**Table 3:** Identification of clusters and area for each cluster in percentage.

Cluster	Area (%)	Sen’s slope (mm year^−1^)	Z_MK_	Pettitt’s Change-point detection (year)
0—Increase	30.9	3.26*	1.53	2007
1—Slight increase	29.28	7.38*	3.25	1998*
2—Slight reduction	31.23	2.18*	1.29	1998
3—Reduction	9.19	− 5.0*	− 2.15	1994*

The Sen’s Slope was calculated with the time series from 1981 to 2020 using the mean of all pixels of each cluster. *Statistically significant at α = 0.05. *Statistically significant at α = 0.05.

All clusters presented statistically significant Z_MK_ trends, with Sen’s Slope values ranging from − 5 to 7.38 mm year^−1^. The Pettitt's Change-point test detected significant changes in clusters 1 and 3, for years 1994 and 1998.

### Mean precipitation for each month

**Figure 6:**
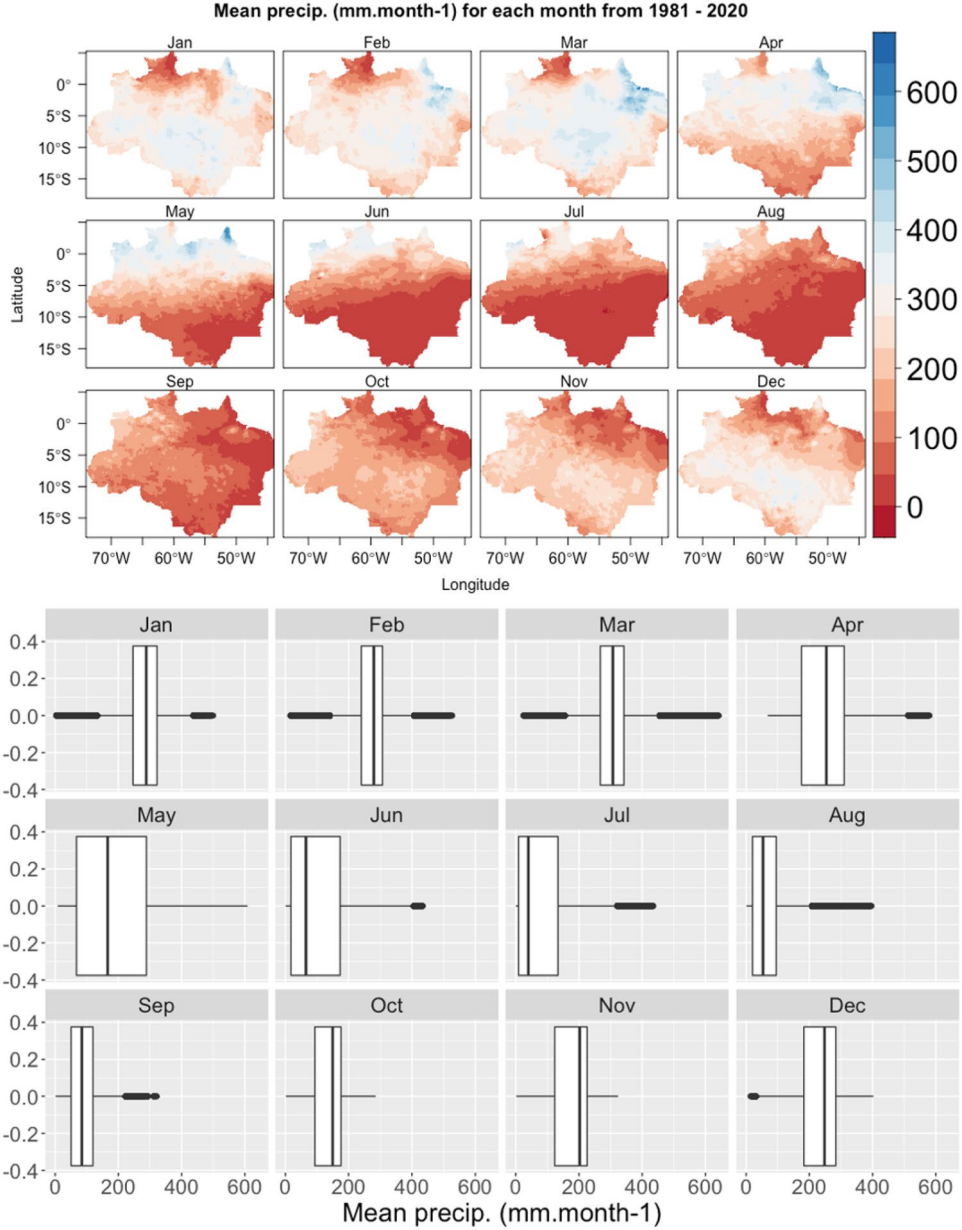
Mean precipitation for each month over the Brazilian Legal Amazon and the boxplots for each month.

Monthly rainfall varied between 0 and over 600 mm. The monthly maps (Fig. 6) show a pattern of spatial distribution of rainfall, with greater concentration in the volume of rainfall in the NW–SE direction during the summer months, relating to the SACZ during this period. In addition to the SACZ, there is also a latitudinal variation in rainfall volumes associated with the ITCZ between the transition months. On the other hand, there is a reduction in rainfall during the winter months due to the ASAS in the areas further south. The driest months are June, July, August and September, with transition periods from October and November (Fig. 6). The wetter months are December, January, February, with transition periods from March to May.

### Sen's slope for each month from 1981 to 2020

**Figure 7:**
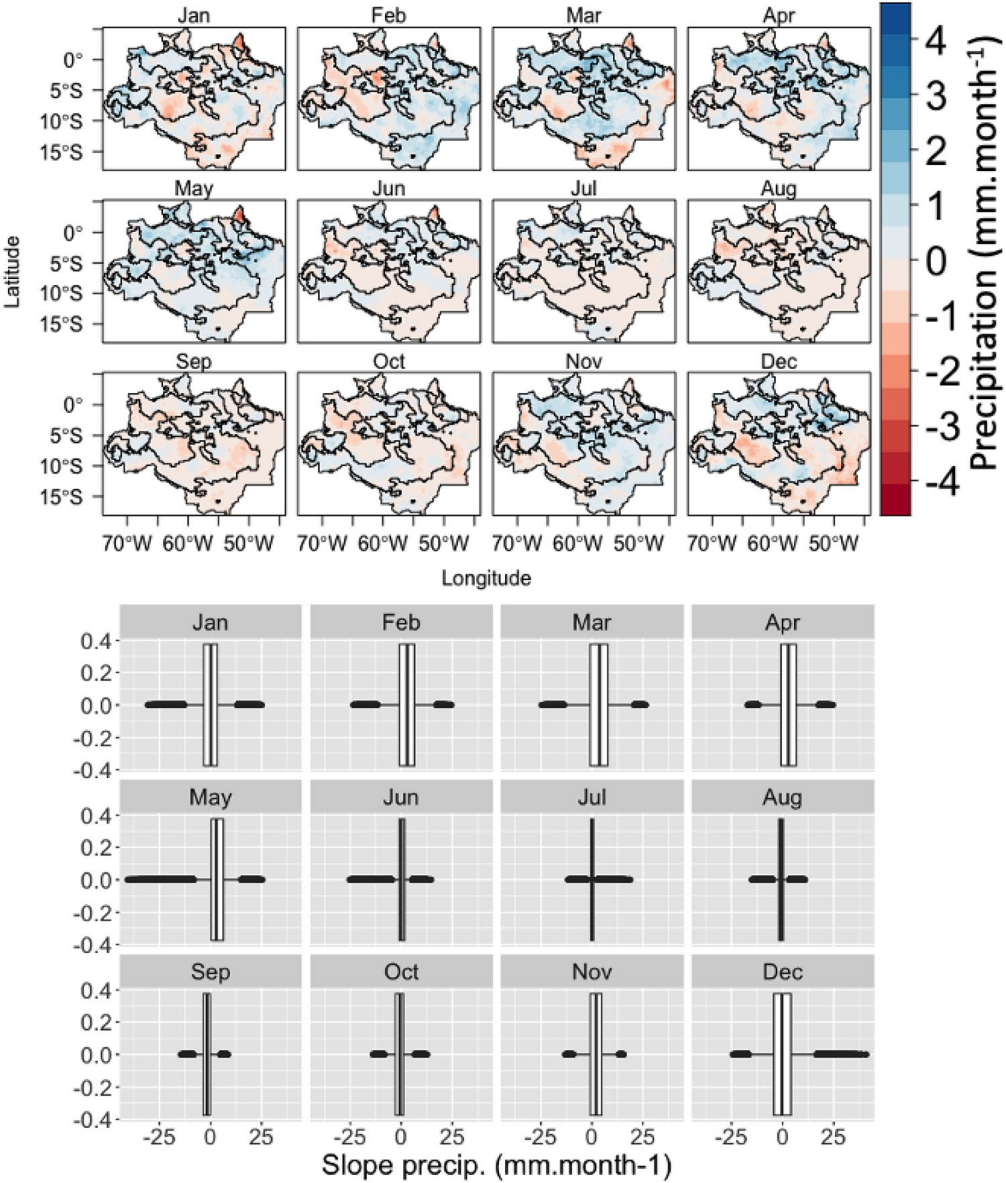
Sen’s Slope of precipitation (mm year^−1^) for each month over the Brazilian Legal Amazon and the boxplots with the distribution of the values for each month. Boundaries of the cluster calculated for the water year from September through August on a time series from 1981 to 2020.

Large regions have increasing rainfall, mainly between the months corresponding to summer and autumn (Fig. 7). Decreasing trends in rainfall (red) were noted in much of the Amazon region, especially during the winter months.

The most humid quarter (D, J, F), had a reduction in rainfall in much of the Amazon region and for the second most humid quarter (M, A, M), there was an increase in rainfall. The monthly variation of the rainfall trends can be seen by the rainfall amplitude (− 2.5 to 2.5 mm month^−1^) displayed in the graphs (bottom) of the figure. Between the months, the summer and autumn periods were the months that presented a greater amplitude in the trends (positive or negative). Between the months corresponding to winter and spring, the amplitude of trends decreases according to the seasonal transition.

Precipitation in the Amazon is influenced by several variables that are part of the hydrological cycle of the Amazon basin. Some of the main variables that influence precipitation in the Amazon include El Niño-Southern Oscillation (ENSO), which is a large-scale climate pattern that affects temperature and precipitation in the Amazon. ENSO is characterized by the warming or cooling of the surface waters of the tropical Pacific Ocean, which in turn affects the atmospheric circulation patterns and the distribution of precipitation. During an El Niño event, the surface waters of the eastern and central Pacific Ocean become unusually warm, while the surface waters of the western Pacific Ocean become cool. This leads to a weakening of the trade winds and a shift in the Inter-Tropical Convergence Zone (ITCZ), where the Northeast and Southeast trade winds meet and where most of the precipitation in the Amazon occurs. As a result, during El Niño events, the ITCZ shifts southward and the precipitation in the Amazon decreases. On the other hand, during La Niña events, the surface waters of the eastern and central Pacific Ocean become cooler than normal, and the trade winds strengthen. This leads to a northward shift of the ITCZ, which enhances the precipitation in the Amazon. So, El Niño events are associated with reduced precipitation in the Amazon, while La Niña events are associated with increased precipitation. This relationship between ENSO and precipitation in the Amazon has been well documented in several studies^31–37^.

### Correlation between precipitation and SST

**Figure 8:**
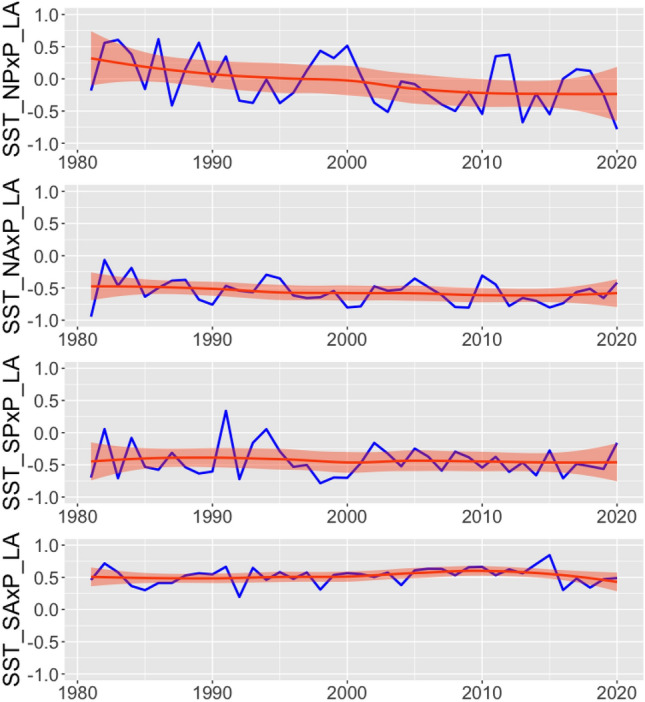
Correlation between SST and the precipitation over the Brazilian Legal Amazon. SST_SA, Sea Surface Temperature for South Atlantic Ocean; SST_SP, Sea Surface Temperature for South Pacific Ocean; SST_NA, Sea Surface Temperature for North Atlantic Ocean; SST_NP, Sea Surface Temperature for North Pacific; P_AL, Precipitation over Legal Amazon.

SST of the Atlantic (north and south) has a more significant correlation with rainfall than the Pacific STT (north and south) (Fig. 8). This influence can be observed in the values shown in the figure (NA-AS), with the northern portion of the Atlantic exerting greater influence in relation to the southern portion of the Atlantic. Our results suggest that in the North Pacific region, there is a relatively weak association or relationship between rainfall and sea surface temperature (SST). However, in comparison to the South Pacific region, this correlation is slightly stronger in the North Pacific when considering the Amazon region.

The correlation between precipitation data and SST changed over time (1980–2020) (Fig. 8). However, in recent decades, a reduction in the correlation on the influence of the South Atlantic has been observed, although a good correlation between rainfall and SST continues. Over time, the Pacific showed a decrease followed by a slight increase in the correlation between SST and rainfall in the Brazilian Amazon region. The northern part of the Pacific has a stronger influence on the Amazon region compared to the southern part.

### Correlation between Tropical Pacific Ocean Sea Surface Temperature and precipitation per cluster

**Figure 9:**
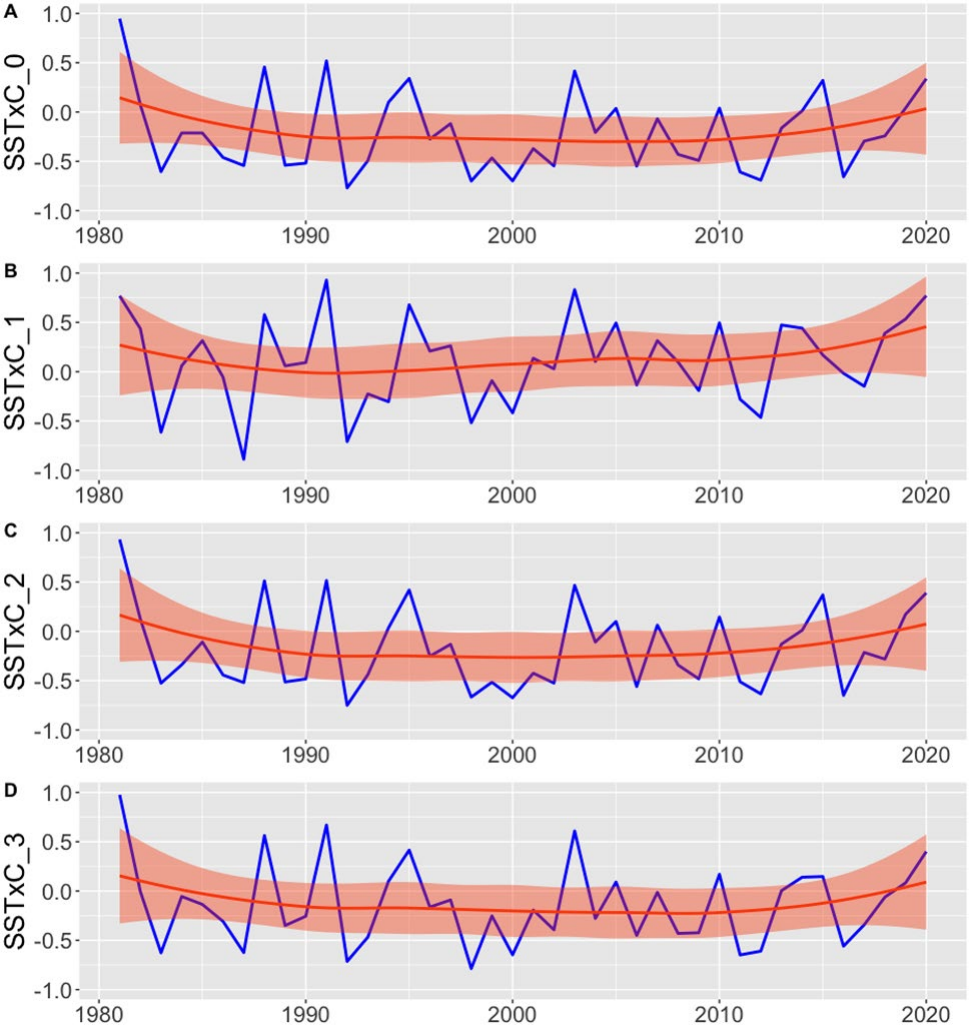
Correlation between the Tropical Pacific Ocean Sea Surface Temperature and precipitation for each cluster.

The correlations vary according to the clusters and the years, representing the spatial and temporal variability of precipitation in the BLA. Among the four clusters (0, 1, 2 and 3) the first presents a more significant correlation than the others, throughout the historical series. The first cluster the correlation between SST and precipitation has been increasing over the decades, that is, this portion of the Amazon has been influenced by TSP (Fig. 9). For the other clusters, a significant influence between TSP and precipitation was not observed over time. Only cluster 0 shows a significant influence of the tropical portion of the Pacific Ocean on the rainfall variability for the Amazon region.

Droughts in the Amazon region during 2005, 2010, and 2015–16 have been linked to elevated sea surface temperatures (SSTs) in the Atlantic Ocean. The relationship between SSTs and Amazon droughts involves complex interactions between oceanic and atmospheric processes. During these drought years, the Atlantic Ocean experienced anomalously high SSTs, particularly in the tropical and subtropical regions. The warmer SSTs create conditions that impact the atmospheric circulation patterns, leading to reduced rainfall in the Amazon rainforest. One key factor is the phenomenon known as the Atlantic Meridional Mode (AMM). The AMM refers to the variation of SSTs in the North Atlantic, characterized by alternating positive and negative phases. During the positive phase of the AMM, the tropical North Atlantic experiences warmer-than-average SSTs. This warmer ocean surface influences the atmospheric circulation patterns, resulting in a weakening of the North Atlantic subtropical high-pressure system. As a result, the trade winds, responsible for bringing moisture to the Amazon region, are weakened. The weakened trade winds reduce the transport of moisture from the ocean to the continent, leading to a decrease in rainfall and the onset of drought conditions in the Amazon. The years 2005, 2010, and 2015–16 coincided with positive phases of the AMM, characterized by elevated SSTs in the North Atlantic. These positive AMM phases contributed to the occurrence and severity of droughts in the Amazon region during those years. This occurs because of the amount of water vapor in the atmosphere. Several studies have shown that there is a positive correlation between Atlantic SST and precipitation in the Amazon basin^32,38^.

Atmospheric circulation plays an important role in the precipitation patterns over the Amazon basin. Two of the most important atmospheric circulation patterns that influence the precipitation in the Amazon can be described with the use of indexes such as the North Atlantic Oscillation (NAO) and the Southern Oscillation Index (SOI). The North Atlantic Oscillation (NAO) is a large-scale atmospheric pressure pattern that varies between positive and negative phases. The positive phase of NAO is associated with a strengthening of the subtropical high-pressure system over the Atlantic Ocean, which leads to a northward shift of the Inter-Tropical Convergence Zone (ITCZ) and increased precipitation in the Amazon. Conversely, the negative phase of NAO is associated with a weakening of the subtropical high-pressure system, which leads to a southward shift of the ITCZ and reduced precipitation in the Amazon. The Southern Oscillation Index (SOI) is a measure of the pressure difference between Tahiti and Darwin, Australia. The SOI is positively correlated with the El Niño-Southern Oscillation (ENSO) phenomenon, which is a large-scale climate pattern that affects temperature and precipitation in many regions around the world. During El Niño events, the SOI is negative, and this is associated with a weakening of the trade winds and a shift in the location of the ITCZ, which leads to reduced precipitation in the Amazon. On the other hand, during La Niña events, the SOI is positive, and this is associated with a strengthening of the trade winds and a northward shift of the ITCZ, which leads to increased precipitation in the Amazon. Studies have shown that the NAO is positively correlated with precipitation in the Amazon basin, while the SOI is negatively correlated^32,39^.

Deforestation in the Amazon basin has been linked to changes in precipitation patterns^10,11,40–42^. Deforestation is the process of clearing natural forests and converting them into other land uses such as agriculture, pasture, or urbanization. This process can have many impacts on the local and regional climate, including changes in precipitation patterns. One of the main ways in which deforestation affects precipitation in the Amazon is through altering the region's energy and water balance. Trees, through transpiration, release water vapor into the atmosphere, contributing to the formation of clouds and precipitation. When forests are cleared, the amount of water vapor released into the atmosphere decreases, which can lead to a reduction in precipitation. Additionally, the removal of trees and vegetation can also change the regional albedo, or reflectivity of the surface, which can affect the amount of solar radiation that is absorbed or reflected by the land surface, and thus the amount of heat energy available to drive the atmospheric circulation and precipitation^43^. Several studies have shown that areas of deforestation in the Amazon experience reduced precipitation compared to nearby forested areas^30,44^. This reduction in precipitation can have negative impacts on the local and regional climate, including changes in temperature and humidity, which can affect the water cycle and availability of water resources for human and ecological needs.

The precipitation variability for each month in the Amazon can vary depending on the specific location within the basin and the year in question. However, in general, the Amazon experiences a distinct wet and dry season. During the wet season, which typically lasts from December to May, the majority of the precipitation in the Amazon occurs. This is due to the northward migration of the Intertropical Convergence Zone (ITCZ), where the Northeast and Southeast trade winds meet and where most of the precipitation in the Amazon occurs. The ITCZ moves northward during this time, which brings increased moisture and precipitation to the region. Additionally, the warmer sea surface temperatures in the Atlantic Ocean during this period can also contribute to increased precipitation. During the dry season, which typically lasts from June to November, precipitation in the Amazon decreases. This is due to the southward migration of the ITCZ, which moves away from the region, and the cooler sea surface temperatures in the Atlantic Ocean during this period, which leads to less moisture and less precipitation.

The tendency of precipitation in the Amazon over time is a topic of ongoing research and debate among scientists. Some studies have found that there has been an overall trend of increased precipitation in the region over the past few decades, while others have found a trend of decreased precipitation.

Other studies have found a trend of decreased precipitation in the Amazon over time. Foley et al.^45^ also used the Sen's slope method and found a decreasing trend of precipitation for the period between 1980 and 2015 in the Eastern Amazon, concluding that the trend in precipitation was not consistent across the entire Amazon, with some areas experiencing increased precipitation while others experienced decreased precipitation. The researchers attributed the decrease in precipitation to the changes in land use, particularly the expansion of agriculture and pasture in the region.

It is important to note that the results of these studies are not necessarily mutually exclusive, and that different regions of the Amazon may be experiencing different trends in precipitation. Additionally, the influence of different drivers such as ENSO, Atlantic SST, atmospheric circulation patterns, and deforestation which can modulate the precipitation patterns, and the human activities, which can cause a deviation from the general pattern.

## Conclusions

In this study we were able to correlate the precipitation of different clusters with SST by regionalizing the trends of increase and decrease in precipitation for the BLA derived from trends using Sen’s Slope using the Google Earth Engine cloud computing platform. In detail, the precipitation in the Amazon region, including the ideal number of clusters, rainfall trends, precipitation time series, monthly precipitation, Sen slope and the influence of SST (Atlantic and Pacific) in the period from 1981 to 2020 has been presented.

Silhouette analysis indicated that the ideal number of clusters is 4, with Display within each cluster similar to each other and dissimilar from other clusters. On the magnitude of the spatially grouped rainfall trends, they showed regions with positive, neutral and decreasing patterns for each cluster. In addition, the precipitation time series for each cluster showed that only one region (cluster 0) presented a different climatic behavior, with an average volume above 200 mm, from the other regions of the Amazon.

The monthly average varies between 0 and more than 600 mm, due to the influence of the seasonality of the atmospheric systems (ITCZ, SACZ and ASAS) that occur in the region. Finally, precipitation trends affected spatial and temporal variations in precipitation in the Amazon region.

Sen's Slope magnitude of trend for each month from 1981 to 2020 showed spatially distributed trends in the Amazon region, showing positive trends and high rainfall amplitude in the summer and autumn months. In the winter period, there were decreasing trends and reduced rainfall amplitude according to the seasonal transition of the Amazonian climate.

On the correlation between precipitation and Sea Surface Temperature (SST) of the Atlantic and Pacific oceans in the Amazon, it was suggested that the North Atlantic SST exerts a more significant influence on rainfall in the Amazon region than in the southern portion. Regarding the Pacific SST, the northern portion has a slight influence, while the southern portion has little influence. Over time, the dynamics between precipitation and South Atlantic SST decreased, while the dynamics between precipitation and Pacific SST increased. When the study was separated into clusters, only cluster 0 showed a significant difference between SST and precipitation in the Amazon region.

In this scenario, the results of this study presented a spatial distribution of the coverage to allow the identification of the areas most accommodated by seasonal changes in patterns compatible with the Amazon region, allowing the understanding of the climatic dynamics and its pluviometric effects for the population. Thus, the information transmitted through this study is important for water management in the Amazon region, allowing the planning of conservation actions, disaster prevention, water resource management, as well as for the development of public policies for the social sectors and showed symptoms in the region.

The findings of this study provide valuable insights for future climate change studies, particularly in relation to the Brazilian Legal Amazon (BLA) region. By identifying distinct precipitation clusters and their correlations with Sea Surface Temperature (SST) patterns in the Atlantic and Pacific oceans, our research offers a framework for understanding the complex interplay between regional climate dynamics and global climate drivers. Researchers conducting future climate change studies can leverage these results to refine models and predictions tailored to the BLA and surrounding areas. Understanding the spatial distribution of precipitation trends and their relationship with SST variations enables more accurate simulations of how future climate scenarios may impact precipitation patterns in the Amazon region. Furthermore, by considering the influence of SST on precipitation at different spatial and temporal scales, future work can better anticipate changes in rainfall regimes, seasonal variability, and extreme weather events, thereby aiding in the development of robust adaptation strategies and policies to mitigate potential risks and promote resilience in the face of climate change.

## Supplementary Information

Supplementary Figures.

## Data Availability

Data will be made available upon reasonable request to the corresponding author, Prof. Rodrigo Moreira, Ph.D., at rodrigo.moreira@unir.br.
