# Supplementary material for: Identifying clusters of precipitation for the Brazilian Legal Amazon based on magnitude of trends and its correlation with sea surface temperature
Source: Sci Rep. 2024 Jun 18;14:14067. doi: 10.1038/s41598-024-63583-x (PMC11718088; doi:10.1038/s41598-024-63583-x)
Supplement: Supplementary file 1 — Supplementary Figures. [file 41598_2024_63583_MOESM1_ESM.pdf]

Supplementary material

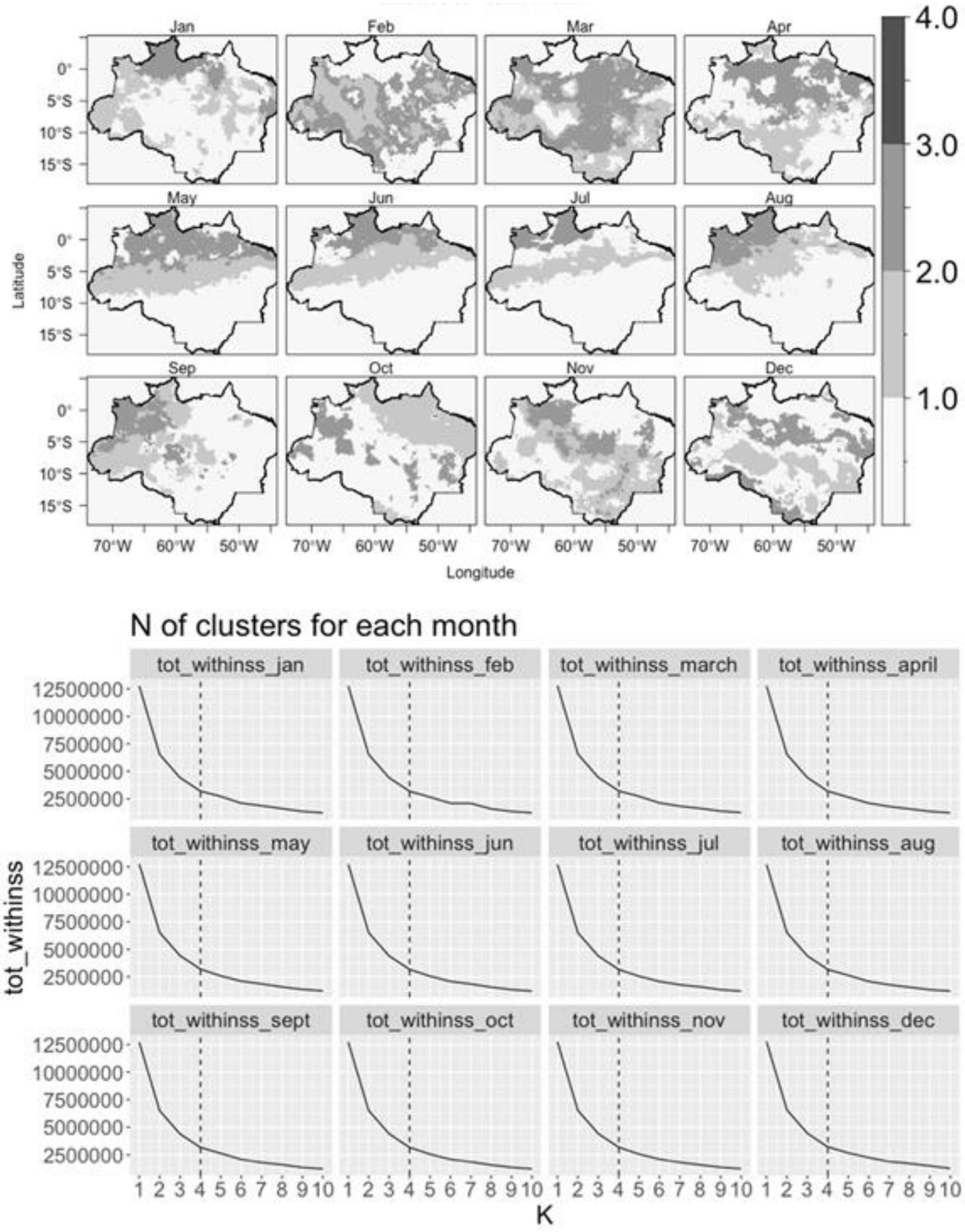

Supplementary material 1. Clusters for each month and the number of clusters identified for each month.

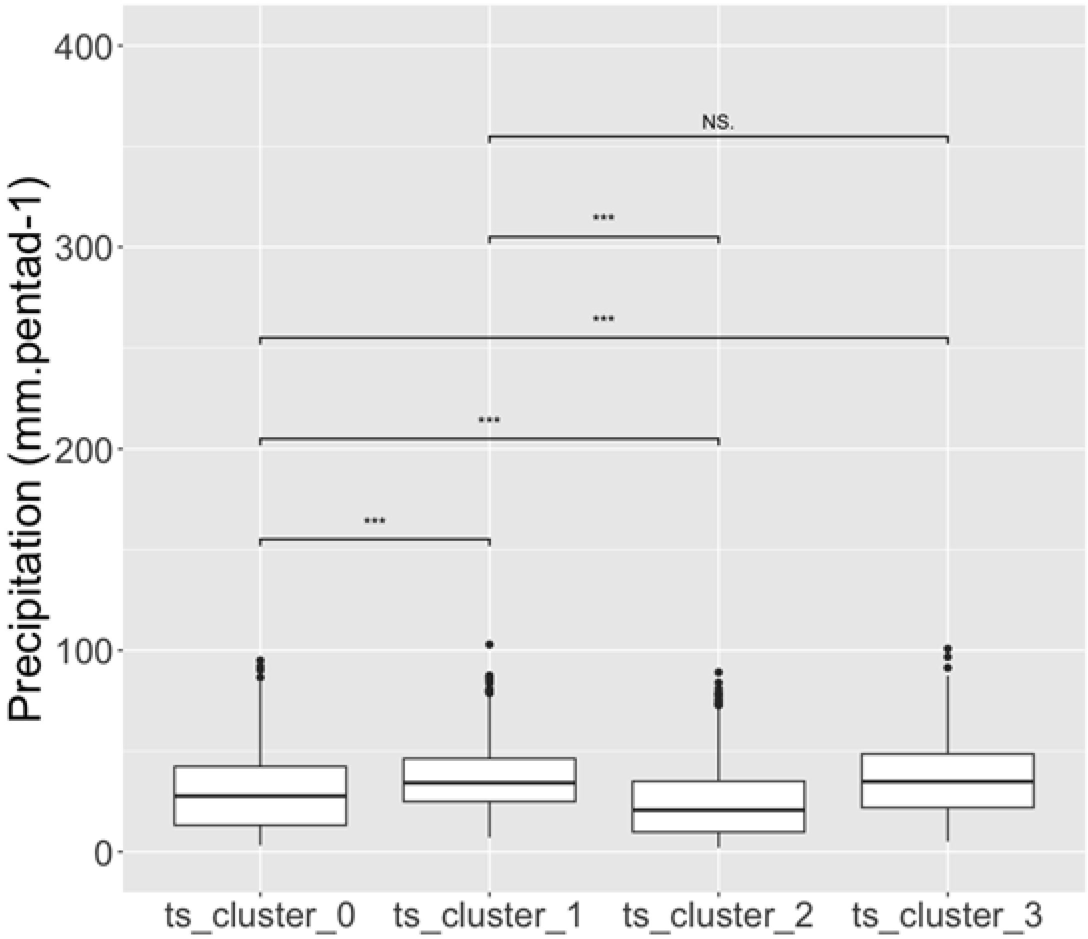

Supplementary material 2. Boxplots of precipitation ($mm.pentad^{-1}$) for each cluster with significance tests ($\alpha = 0.01$).

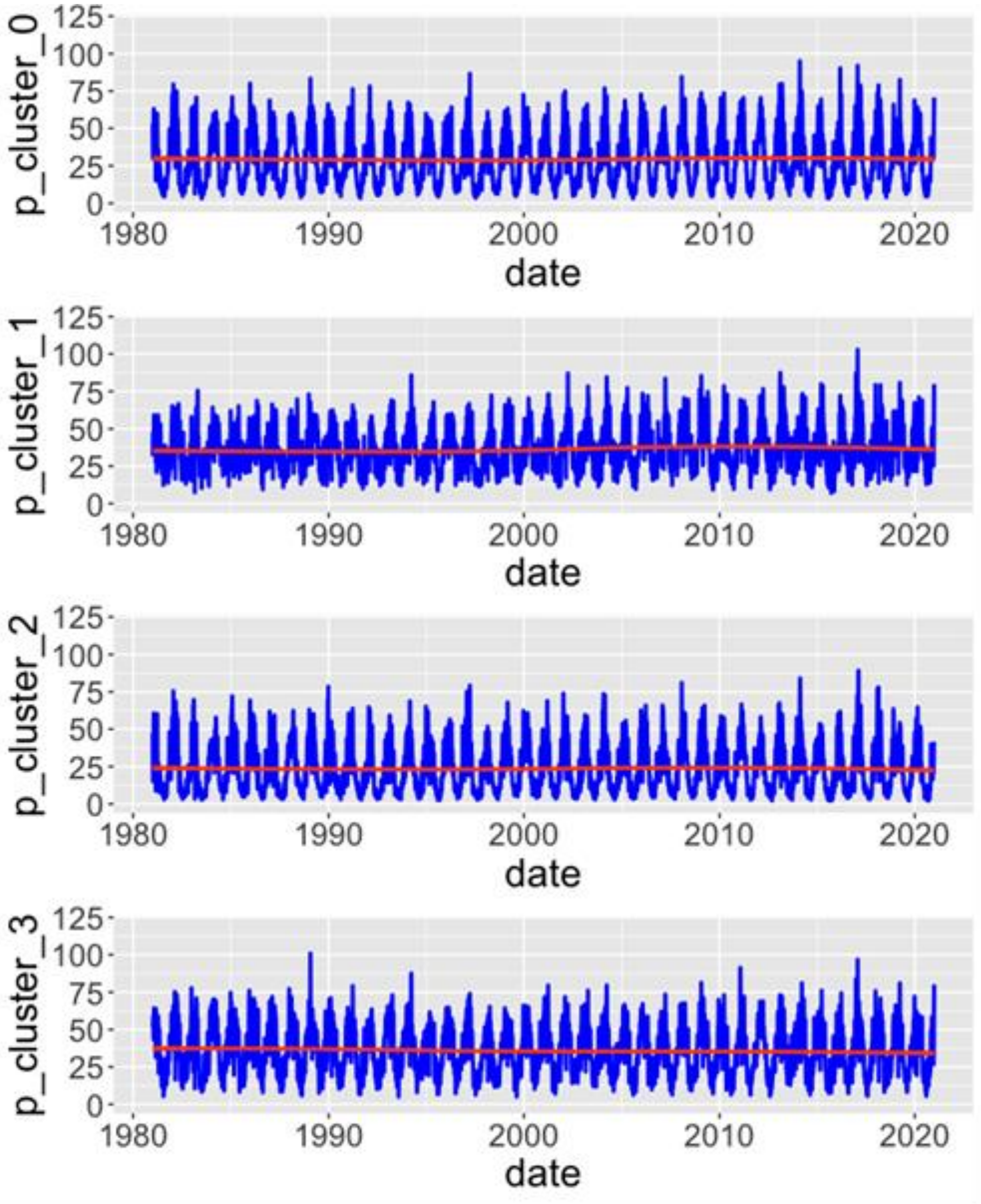

Supplementary material 3. Time series of precipitation ($mm.pentad^{-1}$) for each cluster from 1981 to 2020.
